# Physiological and Differential Proteomic Analysis at Seedling Stage by Induction of Heavy-Ion Beam Radiation in Wheat Seeds

**DOI:** 10.3389/fgene.2022.942806

**Published:** 2022-07-19

**Authors:** Yuqi Li, Jiayu Gu, Ahsan Irshad, Linshu Zhao, Huijun Guo, Hongchun Xiong, Yongdun Xie, Shirong Zhao, Yuping Ding, Libin Zhou, Fuquan Kong, Zhengwu Fang, Luxiang Liu

**Affiliations:** ^1^ College of Agriculture, Yangtze University, Jingzhou, China; ^2^ National Key Facility for Crop Gene Resources and Genetic Improvement, National Center of Space Mutagenesis for Crop Improvement, Institute of Crop Sciences, Chinese Academy of Agricultural Sciences, Beijing, China; ^3^ Biophysics Group, Institute of Modern Physics, Chinese Academy of Sciences, Lanzhou, China; ^4^ China Institute of Atomic Energy, Beijing, China

**Keywords:** ^12^C ion beam, ^7^Li ion beam, proteomics, TMT, wheat

## Abstract

Novel genetic variations can be obtained by inducing mutations in the plant which help to achieve novel traits. The useful mutant can be obtained through radiation mutation in a short period which can be used as a new material to produce new varieties with high yield and good quality wheat. In this paper, the proteomic analysis of wheat treated with different doses of ^12^C and ^7^Li ion beam radiation at the seedling stage was carried out through a Tandem Mass Tag (TMT) tagging quantitative proteomic analysis platform based on high-resolution liquid chromatography-mass spectrometry, and the traditional ^60^Co-γ-ray radiation treatment for reference. A total of 4,764 up-regulated and 5,542 down-regulated differentially expressed proteins were identified. These proteins were mainly enriched in the KEGG pathway associated with amino acid metabolism, fatty acid metabolism, carbon metabolism, photosynthesis, signal transduction, protein synthesis, and DNA replication. Functional analysis of the differentially expressed proteins showed that the oxidative defense system in the plant defense system was fully involved in the defense response after ^12^C ion beam and ^7^Li ion beam radiation treatments. Photosynthesis and photorespiration were inhibited after ^12^C ion beam and ^60^Co-γ-ray irradiation treatments, while there was no effect on the plant with ^7^Li ion beam treatment. In addition, the synthesis of biomolecules such as proteins, as well as multiple signal transduction pathways also respond to radiations. Some selected differentially expressed proteins were verified by Parallel Reaction Monitoring (PRM) and qPCR, and the experimental results were consistent with the quantitative results of TMT. The present study shows that the physiological effect of ^12^C ion beam radiation treatment is different as compared to the ^7^Li ion beam, but its similar to the ^60^Co-γ ray depicting a significant effect on the plant by using the same dose. The results of this study will provide a theoretical basis for the application of ^12^C and ^7^Li ion beam radiation in the mutation breeding of wheat and other major crops and promote the development of heavy ion beam radiation mutation breeding technology.

## Introduction

Common wheat (*Triticum aestivum*) is one of the major cereal crops in the world and its grains contain energy-rich carbohydrates content with useful nutrients and proteins. Mutagenesis plays a significant role in breeding and variety development in cereal (Li et al., 2022). The study of heavy-ion beams had been started in the 1970s in the field of nuclear physics and in 1993 it had been started for plant mutation breeding by using ^12^C and ^7^Li ion beams (Bradshaw, 2017). Recently it has been widely used in cereal crops, especially wheat, rice, and maize for the development of new high-yield varieties (Morishita et al., 2003; Kikuchi et al., 2009; Cabanos et al., 2012; Dong and Li, 2012). The ^7^Li ion beam mutagenesis technology is used for the deletion of susceptible genes, which aids in the understanding of gene function and the development of disease-resistant plants, ultimately accelerating crop improvement (Hase et al., 2012; Fitzgerald et al., 2015).

Both heavy ion beams (^7^Li and ^12^C) and ^60^Co-γ-ray radiation belong to physical agents used for induced mutagenesis. These physical mutagens have been used to create point mutations and small deletions in the genome, as well as DNA methylation variations (Sikora et al., 2011). In contrast, heavy-ion beam-induced mutations are more frequent and more diverse. In addition to point mutations such as single nucleotide base substitutions, insertions, inversions, translocations, and small deletions, ^12^C ion beam radiation can also induce larger deletions, insertions, and chromosomal rearrangements (Shikazono et al., 2005; Nawaz and Shu, 2014). Similarly, ^7^Li ion beam radiation can also induce mutations by disrupting the hydrogen bonds in the DNA double helix structure (Xiong et al., 2020). In plant cells, the mutation-induced by heavy-ion beam radiation is not consistent with the traditional ^60^Co-γ ray radiation.

Proteomics has been widely used in the study of the interaction between cells and the environment, oftenly considered as an effective research method for revealing the regulatory mechanism at the molecular level of cells. For example, the low-temperature resistance of cotton plant is enhanced by increased protein abundance of osmoregulation, cell wall loosening, and cytoskeletal homeostasis (Zheng et al., 2012). Using TMT quantitative protein labeling technology to study the level of enzymes in the theanine synthesis pathway in tea roots under nitrogen deficiency conditions is inhibited, while a large number of enzymes in flavonoid metabolism are up-regulated at the transcriptional level (Wang et al., 2021). According to the proteomic analysis, salt stress increased the accumulation of γ-amino acids in soybean germinated in the dark and improved soybean salt tolerance by the synthesis of reactive oxygen species scavenging enzymes and antioxidants (Yin et al., 2018). Chen et al. (2017) used yeast two-hybrid assay to identify the activation of heat shock protein 21 (HSP21) expression after heat stimulation of *Arabidopsis thaliana*. HSP21 stabilizes the thylakoid structure by interacting with photosystem II at the thylakoid membrane, which contributes to *Arabidopsis thaliana*’s increased heat tolerance (Chen et al., 2017). The analysis of the chloroplast proteome of wheat induced by UV light revealed that differentially expressed proteins were involved in photosynthesis, detoxification, and antioxidant reactions, as well as signal induced transduction pathways and three UV-B protective proteins (Gao et al., 2019). There are many reports available that analyzed protein level change under high temperature, low temperature, drought, high salinity, and UV stress environments, while the changes in proteome caused by ion beam and ray radiation treatments are less studied.

In this study, winter wheat (*Triticum aestivum*) “Heyou 1” and untreated seeds treated with different doses of ^12^C, ^7^Li ion beam, and ^60^Co-γ-ray irradiation were used as materials used for mutagenesis. Proteins contents were extracted from 5-day-old seedlings. The protein level difference between the wheat response to heavy-ion beam and traditional ray radiation was analyzed by the TMT labeling method, which provided a theoretical basis for revealing the regulation mechanism of the wheat response to heavy-ion beam stress.

## Material and Methods

### Plant Materials

Winter wheat (*Triticum aestivum* L.) variety Heyou 1 (HY1) and seeds of HY1were treated with different doses of ^12^C ion beam, ^7^Li ion beam, and ^60^Co-γ ray.

### Radiation Based Mutagenic Treatment

Seeds were irradiated with ^60^Co-γ rays at the Peking University Radiation Center (Beijing, China) at doses of 0, 100, 150, and 250 Gy at a dose rate of 7.5 Gy/min; ^12^C ion beam irradiation was given at the Institute of Modern Physics of the Academy of Sciences (Lanzhou, China) with doses of 0, 40, 60, and 80 Gy, respectively, at a dose rate of 20 Gy/min. Similarly, ^7^Li ion beam radiation treatment was performed at the China Institute of Atomic Energy (Beijing, China) with doses of 0, 25, 50, 75, and 100 Gy at the rate of 8 Gy/min. Each dose was repeated three times with 0 Gy (unirradiated) treated samples as a control.

### Phenotypic Identification and Photosynthetic Parameter Determination

Sixty seeds were taken from each of the 10 radiation-treated and untreated HY1 groups. The seeds were soaked in distilled water at 4 C for 16 h, placed in a germination bag, transferred to a light incubator, and cultured for 7 days at 21 C, 3,000 Lux light, and a light-dark ratio of 16 h:8 h, and the seedling height and root length were measured. The wheat materials used for the determination of photosynthetic parameters were vernalized at 4 C for 35 days, and 30 plants of the treatment group and the control group were transplanted into pots, at a temperature of 20–25 C, a humidity of 50–70%, and supplemented light for 16 h. After culturing for 3 weeks under greenhouse conditions, the photosynthetic parameters of leaves were measured using a MultispeQ instrument (PhotosynQ Inc., East Lansing, MI, United States). One fully expanded leaf was selected for each plant, and the measurement was repeated three times. The data obtained were processed in Excel and plotted with GraphPad Prism8.

### Protein Extraction and Trypsin Digestion

The 5-day old seedlings of the control group and 10 experimental groups were quick-frozen with liquid nitrogen, each group was divided into three biological replicates and completely ground into dry powder, and placed in a 5 ml centrifuge tube. Protein extraction was performed according to Wu (2022). Four times the powder volume of dithiothreitol and protease inhibitor formulated phenol extraction buffer was poured into each group of samples for sonication. The same volume of Tris-equilibrated phenol was poured into a centrifuge tube (4°C, 5,000 g, 10 min). The supernatant was transferred to a new centrifuge tube and 5 volumes of 0.1 M ammonium acetate/methanol were added to precipitate overnight. The final precipitate was reconstituted with 8 M urea, and the protein concentration was determined using BCA Protein Assay Kit (BCA Protein Assay Kit, Beyotime, Shanghai, China). The main process of protein trypsin digestion is that an equal amount of each sample protein is added to an appropriate amount of standard protein, and the pH of the sample is adjusted to about 7.0 with triethylammonium bicarbonate (TEAB). Dithiothreitol (DTT) was added to a final concentration of 5 mM and reduced at 56 C for 30 min 0.5 M iodoacetamide (IAA) was added to the samples to 11 mM and incubated for 15 min at room temperature in the dark. Finally, each sample was diluted with TEAB to a final concentration not higher than 2 M urea. Trypsin was added at a mass ratio of 1:50 (protease: protein) for enzymatic hydrolysis overnight. Then trypsin was added at a mass ratio of 1:100 (protease: protein), and the enzymatic hydrolysis was continued for 4 h. Digestion was terminated by acidifying the samples to pH 3 with trifluoroacetic acid (TFA).

### Analysis of TMT-Labeled Proteins by Liquid Chromatography-Mass Spectrometry

For TMT labeling of peptides followed the method of Wu et al., (2020). The enzymatic fragments were desalted with Strata X C18 (Phenomenex) solid-phase extraction, the column was washed and the samples eluted, and the eluted samples were freeze-dried in the tube. The labeling reagent was taken out from −80°C, placed at room temperature for more than 20 min, the labeling reagent was equilibrated to room temperature, and centrifuged in a mini centrifuge for 3 min. After the peptides were taken out from −20°C, centrifuge at 12,000 g at 4v°C for 3 min, add label buffer to the vortex to dissolve the peptide fragments, and centrifuge at 12,000 g for 3 min at 4°C. Add ACN and vortex to dissolve the TMT reagent, and centrifuge in a mini centrifuge for 5 s. Transfer the TMT reagent to the EP tube containing the peptide fragment, vortex to mix, centrifuge in a mini centrifuge for 5 s, and place at room temperature for 2 h. Each peptide sample was resuspended in 0.5 M TEAB and anhydrous acetonitrile was added. Labeling reagents were added to each corresponding peptide sample at a TMT/peptide ratio of 2:1 (w/w) and incubated for 2 h at room temperature. Each TMT-labeled sample was mixed, desalted, and lyophilized in vacume. The digested peptides were separated by high pH reverse-phase liquid chromatography and separated on an Agilent 300Extend C18 column. The peptide fragments were reconstituted with liquid separation chromatography mobile phase A (0.1% (v/v) formic acid aqueous solvent), and the EASY-nLC 1,000 ultra-high performance liquid phase system was used to separate the peptide fragments at different levels. The separated peptide fragments were ionized into TMT reporter ions using an NSI ion source, which were then analyzed on the Orbitrap Fusion LumosTM mass spectrometer. Peptide precursor ions and their secondary fragments were used with an Orbitrap mass spectrometer. The mass spectrometry proteomics data is deposited to the ProteomeXchange Consortium via the PRIDE partner repository with the dataset identifier ID: PXD033767.

### Database Search

MS data were comprehensively searched using Maxquant (v1.5.2.8). The enzyme digestion method of Trypsin/P was adopted; the number of missed cleavage sites was 2; the minimum length of the peptide was 7 amino acid residues; the maximum number of peptide modifications was set to 5. Cysteine alkylation was set as fixed modification, Oxidation (M), Acetyl (Protein N-term), and Deamidation (NQ) were set as variable modification. The quantitative method was set to TMT-10 plex, and the FDR of protein identification and PSM identification were set to 1%.

### Bioinformatics Analysis for Proteins Characterization

Proteins were annotated using KEGG Automated Annotation Server (KASS) (v.2.0 http://www.genome.jp/kaas-bin/kaas_main). Pathway analysis was performed with KEGG Mapper (V2.5 http://www.kegg.jp/kegg/mapper.html). Subcellular subcellularization of differential proteins were performed by Wolf PSORT (v.0.2 http://www.genscript.com/psort/wolf_psort.html) and CELLO (v.2.5 http://cello.life.nctu.edu.tw/) position (Horton et al., 2007). Perl module was used (v.1.31 https://metacpan.org/pod/Text :: NSP :: Measures :: 2D :: Fisher) Progressive protein function wealth analysis. R Package was used for heat map visualization result (v.2.0.3 https://cran.rproject.org/web/packages/cluster/). For Fuzzy c-means algorithm categorization method, Progressive table categorical analysis, R package Mfuzz Progressive visualization (v.2.32.0 https://www.rdocumentation.org/packages/Mfuzz/versions/2.32.0) was used (Gao et al., 2017). Protein interaction network analysis was performed using Blast, R package networkD3 (v.0.4 https://cran.rproject.org/web/packages/networkD3/), and Cytoscape was used for visualization. cricos diagrams were drawn on the online analysis platform CIRCOS (http://circos.ca/) (Rasche and Hiltemann, 2020).

### Targeted Protein Quantification by Parallel Reaction Monitoring

Refer to the method of Wei et al. (2020) for PRM validation of proteins (Wei et al., 2020). Peptides were ionized and analyzed using Q Exactive™ Plus mass spectrometry. The protease was set to Trypsin (KR/P), the maximum number of missed cleavage sites was set to 0, and the peptide length was set to 7–25 amino acid residues.

### qRT-PCR Validation of Some Differentially Expressed Protein Genes

RNA was extracted using TRNZOL (TIANGEN, China) (Vennapusa et al., 2020). The CDS sequences of the corresponding protein genes were obtained by searching the wheat genome database. Following primers used for qRT-PCR:

TraesCS4A02G116400
  (Forward primer: TTG​TAA​CTA​TCA​AAG​GGT​GCC​AT,
  Reverse primer: CTT​TTA​TTT​CCG​GGC​AAA​ACC​AT).
TraesCSU02G105300
  (Forward primer: TGG​CAT​TCC​ACT​CAA​CTA​CAG​G,
  Reverse primer: ACT​TCA​CAC​CAC​ATG​TAG​GCT​T).
TraesCS3A02G260100
  (Forward primer: CTG​CTA​TAA​CCA​GAG​GCC​GTT​C,
  Reverse primer: TCG​CCA​CGC​CAT​TGT​TAC​AGT).

We performed Quantitative real-time PCR using One-Step gDNA Removal, PerfectStart Green qPCR SuperMix (TransGen Biotech, China), and CFX 96 Real-Time System (Bio Rad, United States). ACTIN was selected as the internal reference gene, and at least three technical replicates were performed for each sample (Zhang et al., 2022).

## Results

### Protein Extraction and Seedling Phenotype After Heavy-Ion Beam Irradiation

**FIGURE 1:**
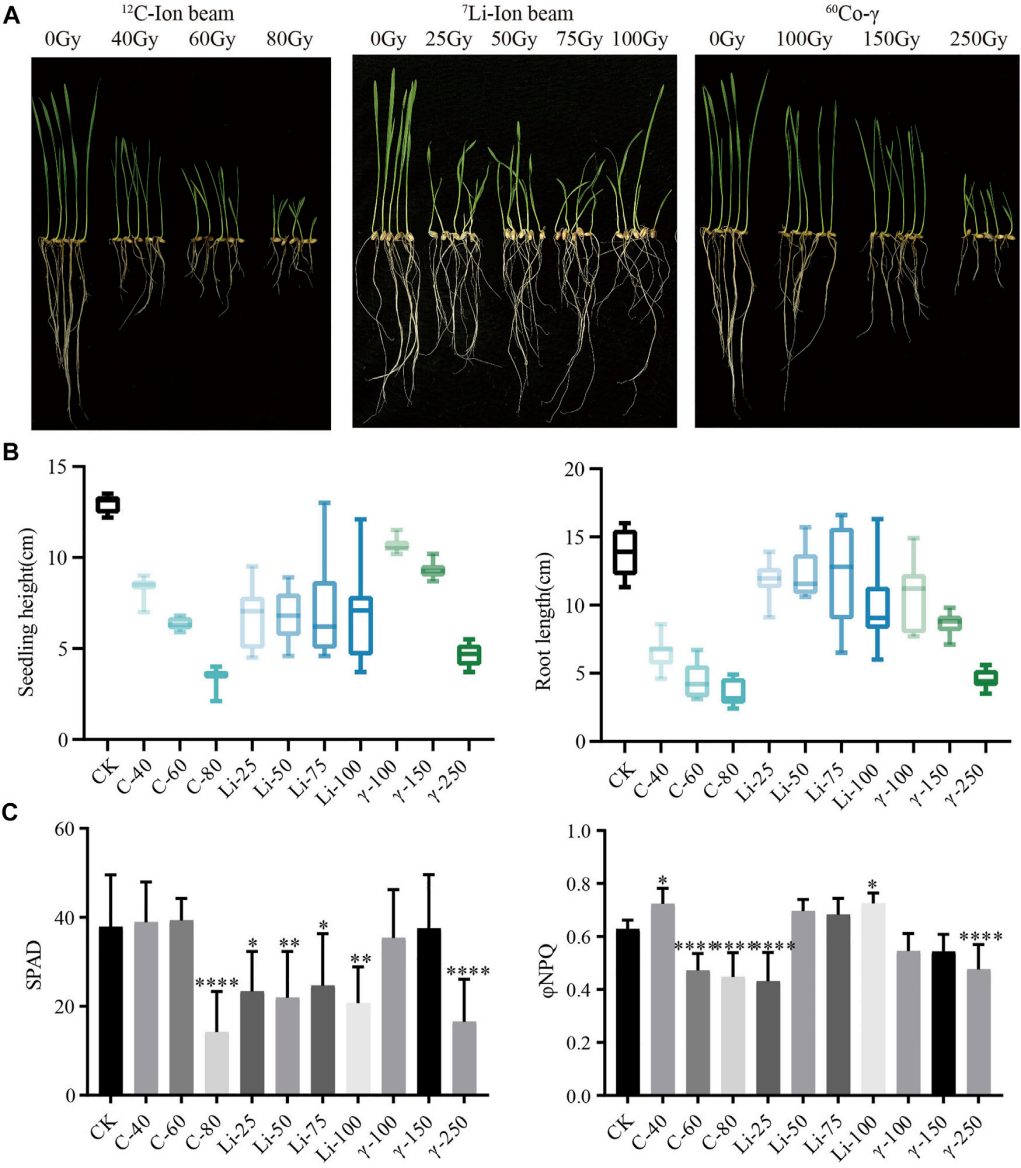
Phenotypic data. **(A)** Growth of HY1 treated with ^12^C ion beam, ^7^Li ion beam and ^60^Co-γ irradiation for 7 days; **(B)** Data of seedling height and root length; **(C)** Photosynthetic parameters SPAD and φNPQ values of 21 days.

HY1 was treated with a^12^C ion beam at 0, 40, 60, and 80 Gy, ^7^Li ion beam at 25, 50, 75, and 100 Gy while ^60^Co-γ ray at 100, 150, and 250 Gy were used for the seedling stage. ^12^C ion beam radiation treatment had inhibitory effects on the seedling height and root length of HY1, and a similar effect had been observed with ^60^Co-γ ray. ^7^Li ion beam irradiation treatment resulted in phenotypic variation such as curling and streak albino in wheat leaves, and all four doses resulted in 100% leaf curling and albino streak (Figures 1A,B). The photosynthesis index showed that the relative chlorophyll content (SPAD) in the high-dose ^12^C ion beam radiation treatment group 80 Gy and the ^60^Co-γ-ray treatment group 250 Gy were significantly lower than those in CK. However, the SPAD values of each dose of ^7^Li ion beam irradiation treatment groups were significantly different from those of CK. The photoprotective chemical quenching index φNPQ of the low-dose ^12^C ion beam irradiation group 40 Gy was significantly higher than that of CK, while the φNPQ value of the high-dose group was significantly decreased (Figure 1C).

**FIGURE 2:**
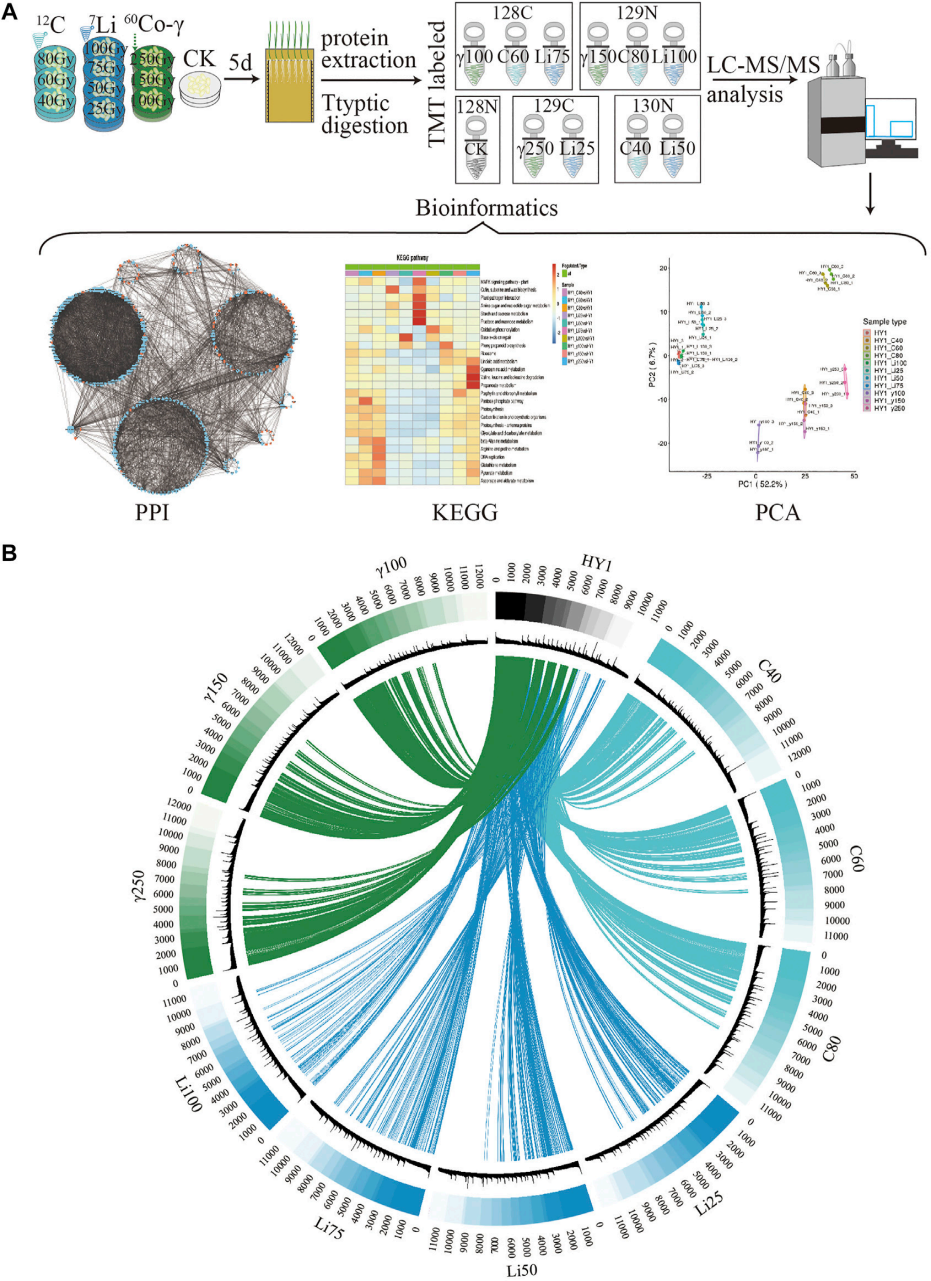
Proteomic Analysis. **(A)** The flow chart of proteomics analysis; **(B)** The Circos diagram (the first circle is the sample name, the second circle is the first-level ranking of the number and depth of proteins, the third circle is the statistics of the occurrences of proteins in each sample group, The darker the color, the higher the counting frequency, the second order of quantitative shared proteins in the fourth circle, and the difference between each radiation treatment group and CK in the fifth circle).

Untreated and treated Heyou-1 5 days seedlings were subjected to protein extraction, identification, mass spectrometry analysis, and bioinformatics analysis such as functional enrichment, expression pattern cluster analysis, and protein interaction analysis (Figure 2A). We performed a macroscopic analysis of the entire proteome in the identified unirradiated and irradiated groups (Figure 2B). Cirocs plots are a collection of protein counts and depths for each treatment sample, the frequency distribution of individual protein counts, quantitative mass spectrometry data for common proteins, and differential protein data for each dose of radiation treatment compared to CK. The amount and depth of protein quantified in the three irradiation-treated HY1 seedlings were inconsistent, the highest in the ^60^Co-γ treatment group was 12,000, the ^12^C ion beam treatment group was between 11,000 and 12,000, and the ^7^Li ion beam treatment group was at 11,000; Deep represents the higher frequency of occurrence in each treatment group; the fourth circle is the second-ranking of the common proteins of each treatment group according to the quantitative information; the fifth circle is the same protein in each treatment group that has a difference in protein expression with the CK group (Figure 2B). By connecting the lines, it was found that there were more differentially shared proteins between the ^12^C ion beam treatment group and the ^60^Co-γ treatment group, while the ^7^Li ion beam treatment group had less differentially shared proteins. However, in the regions with low frequencies of identified proteins, there were more differentially shared proteins between the ^7^Li ion beam treated group and the control (Figure 2B).

### Differentially Expressed Proteins and KEGG Enrichment Analysis

**FIGURE 3:**
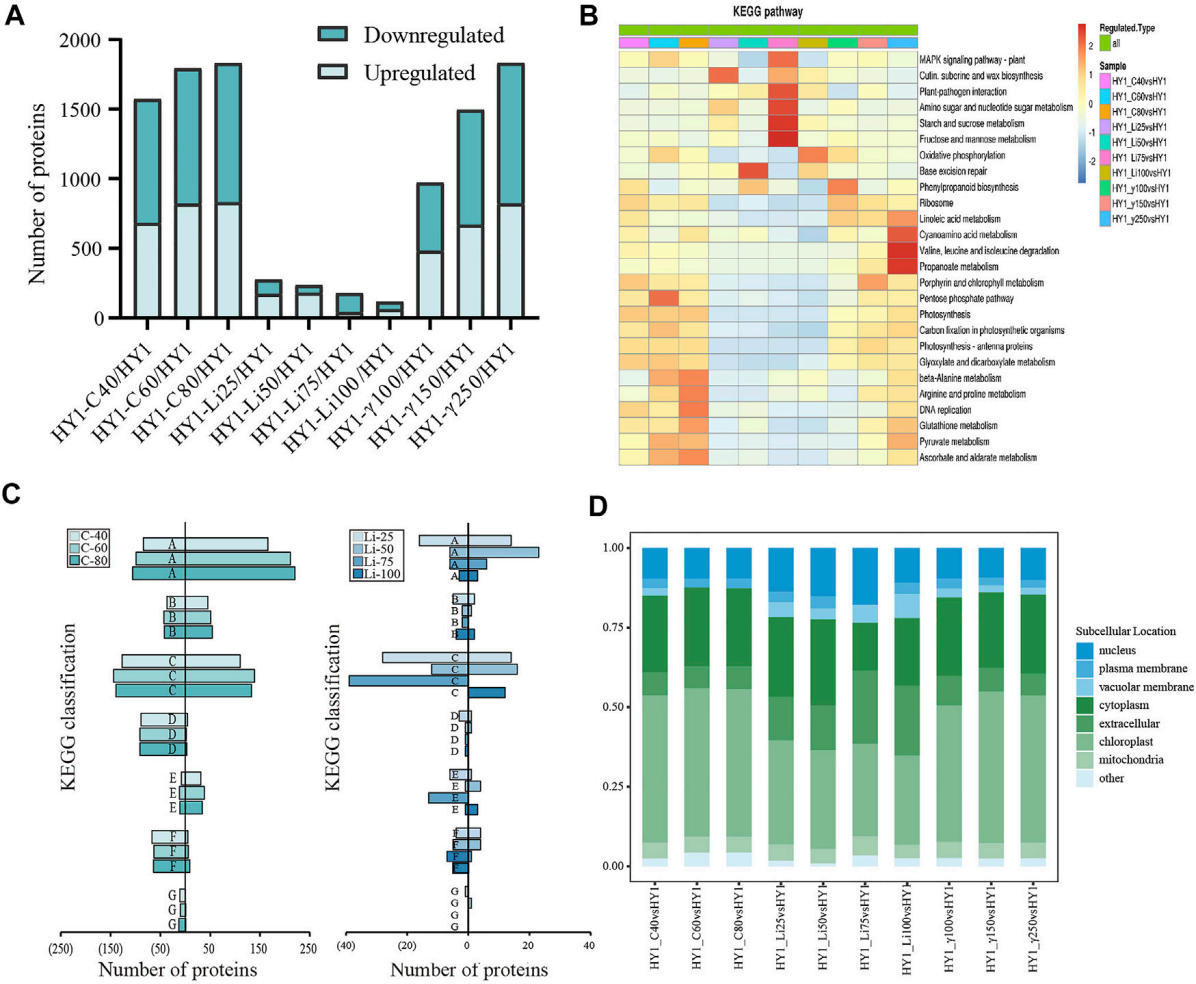
Bioinformatics Analysis. **(A)** Overall map of differentially expressed up-regulated and down-regulated proteins in each dose of radiation treatment group; **(B)** KEGG classification statistics of ^12^C ion beam radiation treatment group, ^7^Li ion beam radiation treatment group and ^60^Co-γ ray radiation treatment group. **(C)** ^12^C ion beam radiation treatment group KEGG classification statistics of down-regulated differential proteins (left side) and up-regulated differential proteins (right side) in radiation treatment group and ^7^Li ion beam radiation treatment group, where A is amino acid metabolism, B is fatty acid metabolism, C is carbon metabolism, D is photosynthesis, E is signal transduction, F is protein synthesis, G is DNA replication. **(D)** Subcellular localization results of each dose of radiation treatment group.

Untreated HY1 was used as the control for the ratio of the expression level of each protein in all 10 treatment groups. Differential expression fold ≥1.3 or ≤ −1.3 as the threshold and *p*-value < 0.05 as the screening criterion, a total of 4,764 up-regulated differentially expressed proteins (DEPs) and 5,542 down-regulated DEPs were obtained in the 10 radiation treatment groups (Figure 3A).

We also analyzed the KEGG enrichment of differentially expressed proteins in different doses of radiation treatment groups, and the differential proteins in the ^12^C ion beam radiation group were significantly enriched in ß-alanine metabolism, arginine, and proline metabolism, DNA replication, ascorbic acid, alginate metabolism and pentose phosphate pathway. Differential proteins in the ^7^Li ion beam irradiation group were significantly enriched in base excision repair, fructose and mannose metabolism, starch and sucrose metabolism, amino sugar and nucleotide sugar metabolism, and plant-pathogen response. Similarly, differential proteins in the ^60^Co-γ irradiation group were significantly enriched in valine, leucine, and isoleucine metabolism, cyano amino acid metabolism, and propionate metabolism (Figure 3B).

We broadly divided the enriched KEGG pathways into seven types for analysis (amino acid metabolism (A), fatty acid metabolism (B), carbon metabolism (C), photosynthesis (D), signal transduction (E), protein synthesis (F) and DNA replication (G)). The proteins up-regulated for ^12^C ion beam radiation treatment was mainly concentrated in the KEGG pathway of A, B, C, and E types, with a high proportion of A and C types. The down-regulated proteins were mainly concentrated in the KEGG pathway of A, C, D, F, and G types. The up-regulated proteins after ^7^Li ion beam irradiation were mainly concentrated in the KEGG pathways of types A and C, while the down-regulated proteins are mainly concentrated in the KEGG pathways of types C, E, and F. The number of proteins in each KEGG pathway was less, and there was no difference in some pathways (Figure 3C).

For further understanding of the intracellular distribution of the identified proteins, subcellular localization analysis was also performed. The subcellular localization of the differentially expressed proteins in the ^12^C ion beam and ^60^Co-γ-ray irradiation groups was mainly in the chloroplast and cytoplasm, while the proportion of the differentially expressed proteins for the ^7^Li ion beam showed less in the chloroplast and cytoplasm (Figure 3D).

### Co-Clustering Analysis of Differential Proteins in Groups Treated With Heavy Ion Beam and Gamma-Ray Irradiation

**FIGURE 4:**
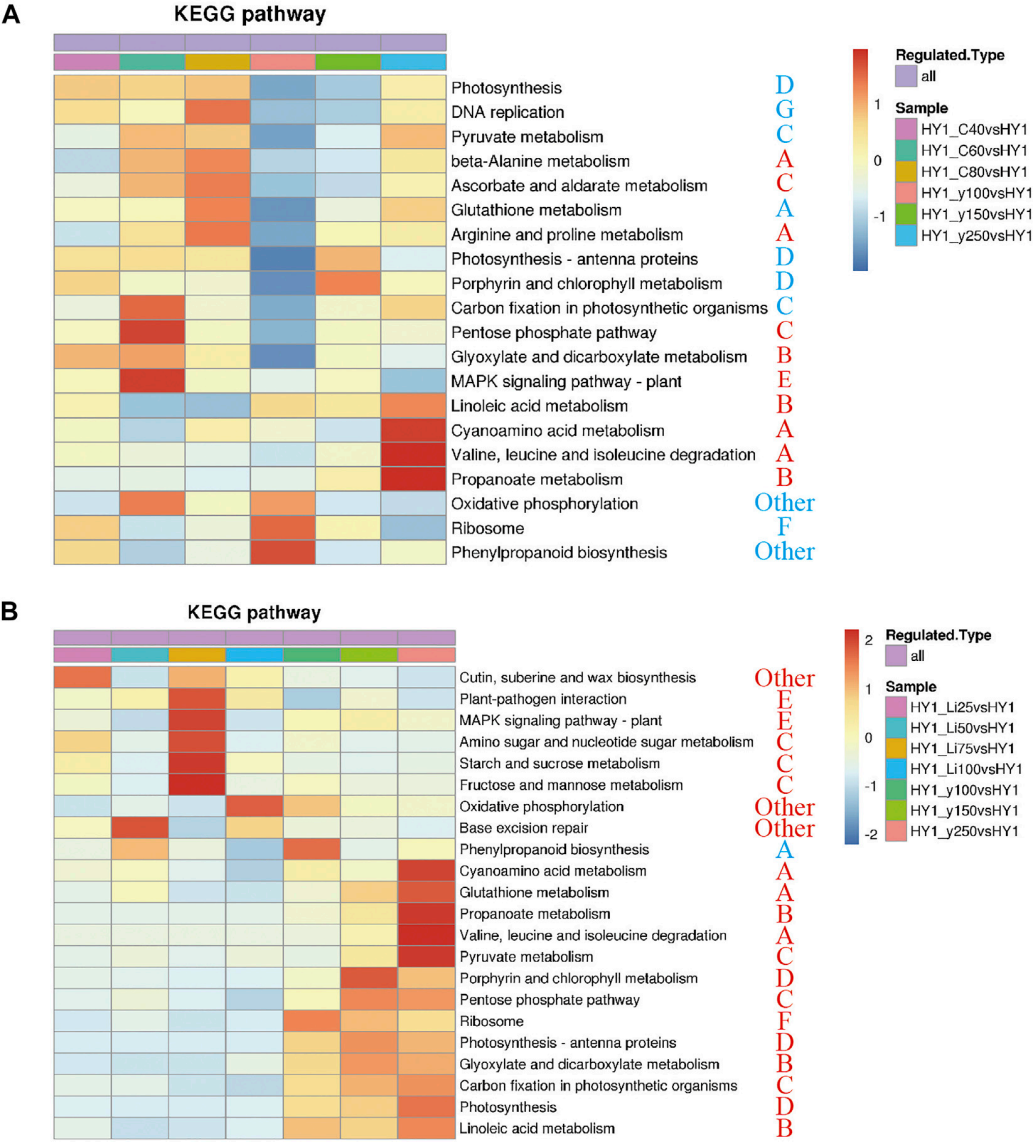
KEGG heat map. **(A)** KEGG heat map of ^12^C ion beam radiation treatment group and ^60^Co-γ-ray radiation treatment group. **(B)**co-clustering KEGG heat map of ^7^Li ion beam radiation treatment group and ^60^Co-γ-ray radiation treatment group.

Co-clustering analysis of ^12^C ion beam and ^60^Co-γ-ray irradiation treatment group together enriched 10 KEGG pathways including A, C, D, G, F, and D types (“photosynthesis”). Among the 10 differential pathways, the ^12^C ion beam irradiation group was significantly clustered in the “pentose phosphate pathway” (C) and the “MAPK signaling pathway” (E), while the ^60^Co-γ-ray treatment group was significantly clustered in the “valine, leucine and “Isoleucine metabolism” (A), “cyanoamino acid metabolism” (A) and “propionate metabolism” (B) (Figure 4A). The 21 differential pathways included A, B, C, E and F types, the ^7^Li ion beam irradiation group was enriched to 8 KEGGs including “starch and sucrose metabolism” (C) and “MAPK signaling pathway” (E), while ^60^Co-γ-ray treatment group was enriched in 13 pathways including “propionate metabolism” (B), “porphyrin and chlorophyll metabolism” (D) (Figure 4B).

### Cluster Analysis of Differentially Expressed Protein

**FIGURE 5:**
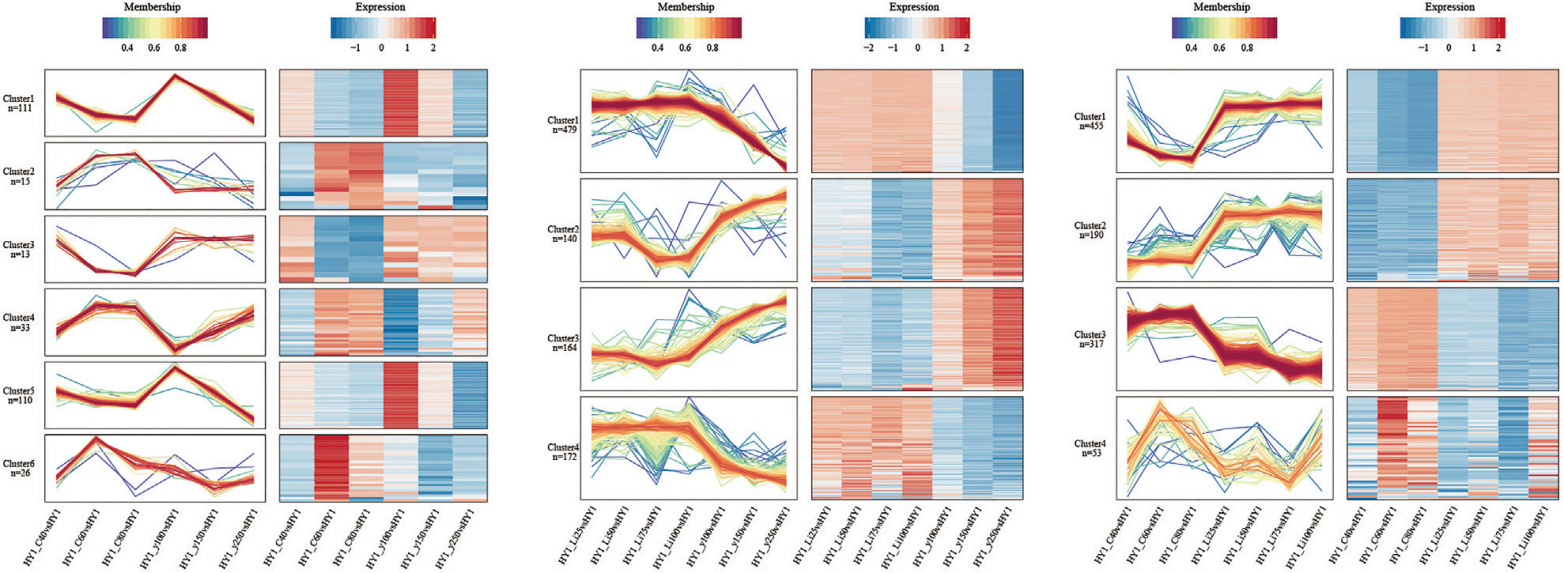
Expression pattern clustering and protein network interaction map. From left to right are the cluster analysis of ^12^C ion beam and gamma ray treatment comparison group, ^7^Li ion beam and gamma ray treatment comparison group, and carbon ion beam and ^7^Li ion beam beam treatment comparison group.

In this study, the Mfuzz method was used to perform cluster analysis on protein abundance transformation under different consecutive samples, including the comparison group of ^12^C ion beam and ^60^Co-γ ray irradiation treatment (I), and the comparison group of ^7^Li ion beam and ^60^Co-γ ray irradiation treatment group (II). Similarly, a comparison group (III) of ^12^C ion beam and ^7^Li ion beam irradiation treatments (Figure 5).

The comparison group consists of 6 clusters. The number of proteins in cluster 1 and 5 were higher and the trend of protein abundance decreased with increasing dose in both ^12^C ion beam and ^60^Co-γ-ray irradiation groups, and the KEGG pathway was significantly enriched in photosynthesis and carbon metabolism. The changing trend of protein abundance in cluster 4 was different from that of cluster 1 and 5, and the KEGG pathway was significantly enriched in amino sugar and nucleotide sugar metabolism (C), MAPK-signaling pathway (E), and phytohormone signal transduction (E). The changing trends of protein abundances in Clusters 2, 3, and 6 were opposed to all other clusters for ^12^C ion beam and ^60^Co-γ-ray irradiation, and the KEGG pathway was enriched to arginine metabolism (A), glycerophosphate metabolism (B) and Protein trafficking in the endoplasmic reticulum (F).

There were 4 clusters in comparison group II. The protein abundance in clusters 2 and 3 decreased with increasing dose after ^7^Li ion beam irradiation and increased with increasing dose after ^60^Co-γ ray irradiation. The KEGG pathway was enriched to glutamate metabolism (A), amino sugar and nucleotide sugar metabolism (C), and phytohormone signaling (E). The trend of protein abundance in clusters 1 and 4 was opposite to that in clusters 2 and 3, and the KEGG pathway was enriched in photosynthesis (D), carbon fixation (C), and ribosomes (F).

In comparison to group III, 4 clusters were also developed. The protein abundances in clusters 1 and 2 decreased with increasing dose after ^12^C ion beam irradiation and increased with increasing dose after ^7^Li ion beam irradiation. The KEGG pathway was enriched to photosynthesis (D), ribosomes (F), and carbon fixation (C). The changing trend of protein abundance in Clusters 3 and 4 is opposite to that in clusters 1 and 2. The KEGG pathway is enriched to phenylpropane synthesis (A), amino sugar and nucleotide sugar metabolism (C), and MAPK-signaling pathway (E).

### Protein Interaction Analysis

The interaction analysis of differentially expressed proteins was carried out for each dose of ^12^C and ^7^Li ion beams irradiation treatment groups. The results showed that the ^12^C ion beam radiation treatment group had more interactions between protein synthesis and transport-related proteins with relatively more interactions between photosynthesis-related proteins and carbon metabolism-related proteins (Supplementary Figure S1). In the ^7^Li ion beam irradiation group, there were fewer interactions among different metabolisms with fewer PPI interactions, and only two interactions (between signal transduction and protein synthesis; between amino acid metabolism and carbon metabolism) (Supplementary Figure S2).

### PRM and qRT-PCR Validation Results

**FIGURE 6:**
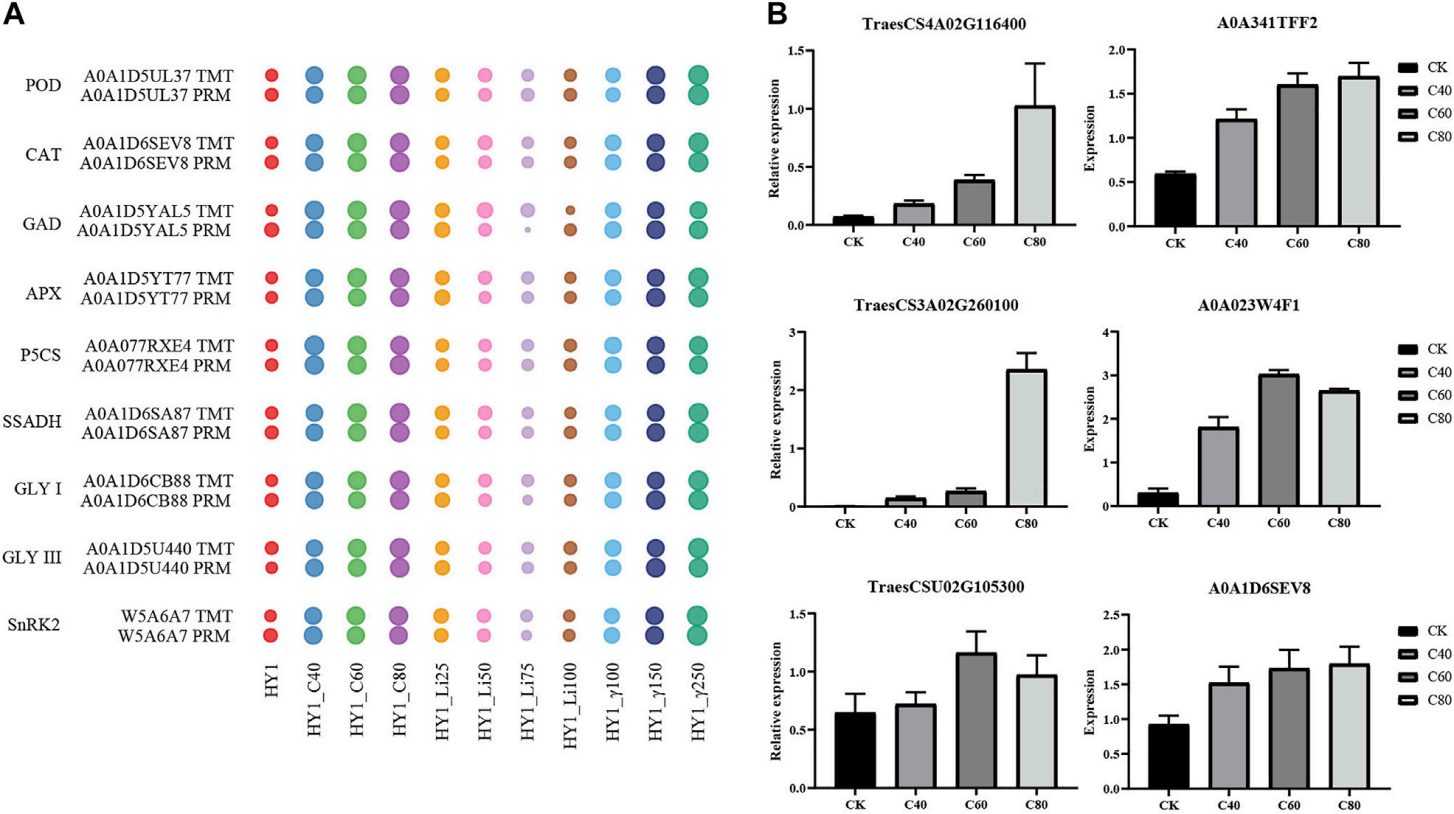
PRM verification. **(A)** Water drop diagram of quantitative comparison results between TMT and PRM of 9 proteins. **(B)** qRT-PCR results of the corresponding genes of three differentially expressed proteins.

Mass spectrometry-based targeted protein validation (PRM) is a high-resolution, high-precision mass spectrometry-targeted quantification technology that achieves relative or absolute quantification of target proteins/peptides through selective detection of target proteins/peptides. We selected A0A1D5UL37, A0A1D6SEV8, A0A1D5YAL5, A0A1D5YT77, A0A077RXE4, A0A1D6SA87, A0A1D6CB88, A0A1D5U440, and W5A6A7 from 10 groups of 10306 differentially expressed proteins for PRM validation. In each dose of ^12^C ion beam, ^7^Li ion beam irradiation treatment group and 60Co-γ ray treatment group, the TMT and PRM results of the changes in the abundance of these 9 proteins were consistent (Figure 6A). We selected three differential proteins, including the disease process-related protein (A0A341TFF2), chitinase (A0A023W4F1) and catalase (A0A1D6SEV8) corresponding genes TraesCS4A02G116400, TraesCS3A02G260100 and TraesCSU02G105300 for qRT-PCR verification. The expression levels of three protein-coding genes increased in the 40 Gy, 60 Gy, and 80 Gy ^12^C ion beam treatment groups, and the results were consistent with the data obtained by proteomic analysis (Figure 6B).

### qRT-PCR Results of Differentially Expressed Protein Genes

We selected three differential proteins, including the disease pathogenesis-related protein (A0A341TFF2), chitinase (A0A023W4F1) and catalase (A0A1D6SEV8) corresponding genes TraesCS4A02G116400, TraesCS3A02G260100 and TraesCSU02G105300 for qRT-PCR verification. The expression levels of three protein-coding genes increased, and the results were consistent with the data obtained by proteomic analysis (Supplementary Figure 3).

## Discussion

### Plant Defense Systems Are Fully Mobilized in Response to Radiation Treatment

**FIGURE 7:**
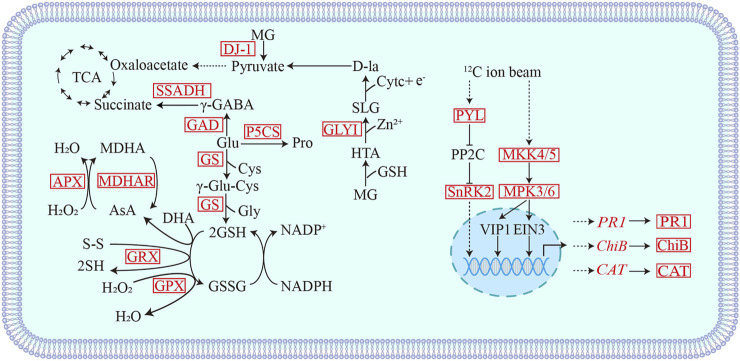
Schematic diagram of differential proteins involved in heavy ion beam radiation stress response. MG: Methylglyoxal; HTA: Hemithioacetal; SLG: S-d-lactosylglutathione; SSADH: Succinic semialdehyde dehydrogenase; GAD: Glutamate decarboxylase; GS: Glutamine synthase; GRX: Glutaredoxin; GPX: Glutathione peroxidase; APX: Ascorbate peroxidase; MDHAR: Monodehydroascorbate reductase; GLYI: Acetalase I; DJ-1: GLY III; PYL: ABA receptor; SnRK2: SNF1-related protein kinase; PR-1: pathogenesis-related protein; MAKK5: Mitogen-activated protein kinase5; ChiB: chitinase; CAT: Catalase. Red indicates differentially up-regulated proteins.

Generally, when plants signalled abiotic stress, a complex, and efficient defense systems are activated, such as enzymatic antioxidants, non-enzymatic antioxidants, osmotic regulators, and glyoxalase systems (Kim et al., 2010). Stresses such as high salt, drought, UV radiation, heavy metals, and extreme temperatures will eventually led to oxidative stress, increasing the content of antioxidant enzymes, such as catalase (CAT), peroxidase (POD) in antioxidant defense mechanisms, superoxide dismutase (SOD) (Gill and Tuteja, 2010; Anjum et al., 2016). In our study, the proteomic data of the ^12^C, ^7^Li ion beam, and ^60^Co-γ-ray radiation-treated groups, the main antioxidant systems involved in the radiation response included the antioxidant GSH, glutamate metabolism, and the aldolase system (Figure 7). Glutamine synthase (GS) catalyzes the production of GSH from glutamylcysteine and glycine (Vaish et al., 2020). Glutathione peroxidase (GPX) and glutaredoxin (GRX) oxidize H_2_O_2_ and disulfide (R-S-S-R′) to (H-S-S-R), H_2_O, and GSSG in the presence of GSH (Xiao et al., 2019). The ascorbic acid-glutathione cycle (AsA-GSH) plays the role of “core of redox signaling” in stress-threatened plants and is involved in key processes of hydrogen peroxide metabolism, and its key enzymes include ascorbic acid peroxide (APX) and monodehydroascorbate reductase (MDHAR) (Foyer and Noctor, 2011; Tanaka et al., 2021). In response to radiation, wheat seedlings scavenge H_2_O_2_ and disulfide P (SSG) through GSH-dependent GPX, GRX, and GS, as well as the ascorbic acid-glutathione cycle. Glutamate (Glu) metabolism also plays an important role in stress resistance. Under the action of glutamate decarboxylase (GAD) and delta-1-pyrroline-5-carboxylic acid synthase (P5CS), glutamate is converted into antioxidants gamma-aminobutyric acid (GABA) and proline, respectively (Pro), maintains intracellular redox homeostasis (Kaur and Asthir, 2015). GABA in turn generate succinate under the action of succinate semialdehyde dehydrogenase (SSADH) and return to the TCA cycle (Hijaz et al., 2018). The expressions of GAD, P5CS, and SSADH were up-regulated after irradiation, indicating that multiple antioxidant pathways related to glutamate metabolism were also involved in the oxidative stress response induced by radiation. In addition, aldolase I (GLY I) and DJ-1 (GLY III) were differentially upregulated in the aldolase detoxification system. The GSH-dependent aldolase detoxification system consists of GLY I and GLY II, and the independent pathway is a shorter, metal-independent and GSH-independent pathway, which is acted by the DJ-1 protein (Banerjee et al., 2020). Methylglyoxal (MG), which is easy to form toxic substances, is converted into pyruvate by the acetylase system and enters the TCA cycle, and is reused (Bhowal et al., 2020).

We selected representative proteins related to antioxidant effects after radiation treatment for PRM verification, including the above-mentioned POD, CAT, GAD, APX, P5CS, SSADH, GLY I, and GLY III. The expression changes of these proteins were correlated with the quantitative results of TMT. consistent (Figure 6A).

### The Effects of ^12^C and ^7^Li Ion Beam Irradiation Treatments on Photosynthesis and Photorespiration Were Different

Photosynthesis is the most basic and complex physiological process in plants, including photosynthetic pigments and photosystem, electron transport system, and CO_2_ reduction pathway, so damage caused by any level of stress may reduce the overall photosynthetic capacity of green plants (Ashraf and Harris, 2013). For example, UV-B irradiation reduces photosynthetic pigments in wheat and damages photosystem response center proteins (Zu et al., 2004; Qiu et al., 2007; Joshi et al., 2011; Henderson et al., 2013). Low-dose stress maintains a higher concentration of chlorophyll to tolerate the stress, while high-level stress inhibits the synthesis and accumulation of chlorophyll (Agathokleous et al., 2020). In addition, the photorespiration cycle interacts with photosynthesis and amino acid metabolism and also functions to remove toxic metabolites. Research showed that photorespiration is inhibited under high temperature, strong light, drought, and salt stress (Liu et al., 2019; Timm et al., 2019).

Our results showed that ^12^C ion beam and ^60^Co-γ-ray irradiation treatment resulted in down-regulated expression of proteins related to photosynthesis and photorespiration, while the ^7^Li ion beam irradiation treatment group had no difference. For example, photosystem I, photosystem II, and electron carriers are involved in the light reaction in photosynthesis, ribulose-1,5-bisphosphate carboxylase (Rubisco) involved in the dark reaction, and NADPH-pro, a key enzyme in chlorophyll synthesis. Chlorophyll oxidoreductase (POR) and protoporphyrinogen IX oxidase (PPO) were differentially downregulated. Glycerate-3-kinase (GLYK), ferredoxin-dependent glutamate synthase (GOGAT), CAT, glutamate: glyoxylate aminotransferase (GGT), glycine decarboxylase (GDC), and serine hydroxymethyltransferase (SHMT) were differentially downregulated. The light and dark reactions of photosynthesis and photorespiration were inhibited after ^12^C ion beam and ^60^Co-γ-ray irradiation treatment, which slowed down the growth rate of wheat seedlings, showing that the damage rate of seedling height and root length increased with the increase of treatment dose. Increased dose effect. However, ^7^Li ion beam treatment had less effect on photosynthesis and photorespiration, and the physiological damage effect on wheat seedling growth was different from ^12^C ion beam and ^60^Co-γ-ray irradiation.

### The Synthesis of Biological Macromolecules Such as Proteins Is Affected by Radiation

The heat shock protein family consists of constitutive and stress-inducible types, including small HSP, HSP40, HSP70, HSP90, and their related molecular chaperones (Kampinga et al., 2009). HSP/chaperones are major components of multiple stress responses, of which HSP70 and HSP90 are involved in signal transduction, protein targeting, and degradation (Jacob et al., 2017). Under heat stress conditions, HSP70 interacts with phospholipase to regulate phospholipid metabolism, while HSP90.1 plays a role in plant heat tolerance by interacting with autophagy receptors to mediate degradation (Song et al., 2021; Thirumalaikumar et al., 2021). Our results indicated that multiple heat shock proteins were differentially expressed in the three radiation-treated groups, including HSP90, HSP70, HSP83, HSP26, smHSP, and smHSP24.1, smHSP23.6, smHSP23.2, smHSP 22.3, and HSP17.9. Therefore, protein quality control plays an important role in the recovery of wheat seedlings after radiation stress.

### ^12^C Ion Beam Radiation Treatment Induces Activation of the MAPK Signaling Pathway

The mitogen-activated protein kinase cascade (MAPK cascade) acts downstream of receptors/signal receptors to coordinate cellular responses for normal plant growth and development, immune responses, and responses to abiotic stresses (Meng and Zhang, 2013; Xu and Zhang, 2015; Zhang et al., 2018). MKK4/5 activates MPK3/6 in MAPK signaling (Cai et al., 2014; Zipfel, 2014; Langner and Gohre, 2016; Giese et al., 2018). In addition, UV-B, heat stress, etc. can activate MPK3/6 signaling, act on the downstream transcription factor EIN3 to induce the accumulation of chitinase or activate the expression of disease process-related proteins (PR) and CAT-encoding genes (Rakwal et al., 2004; Yoo et al., 2008; Bethke et al., 2009; An et al., 2010; An et al., 2021; Kumar et al., 2021). ^12^C ion beam radiation treatment resulted in differential upregulation of mitogen-activated protein kinase 3 (MPK3), mitogen-activated protein kinase 5 (MKK5), disease process-related protein (PR1), chitinase (ChiB) and CAT, while ^7^Li PR1 and ChiB were differentially downregulated in the ion beam treated group. We selected *PR1* (*TraesCS4A02G116400*), *ChiB* (*TraesCS3A02G260100*) and *CAT* (*TraesCSU02G105300*) genes for qRT-PCR validation (Figure 6B). The expression of PR, ChiB, and CAT genes increased in the ^12^C ion beam irradiation group, suggesting that the accumulation of PR, ChiB and CAT proteins may be the result of transcriptional regulation (Figure 7).

In addition, SNF1-related protein kinase (SnRK2) is a positive regulator in the ABA signal transduction pathway, and the ABA receptor PYL activates SnRK2 by blocking the action of its inhibitor PP2C and regulates the expression of downstream ABA-related defense genes (Gong et al., 2020; Maszkowska et al., 2021). We also selected the SnRK2 protein involved in signal transduction for PRM verification, which was consistent with the quantitative results of TMT. Therefore, SnRK2 and ABA receptor (PYL) were differentially up-regulated in the ^12^C ion beam radiation treatment group, which may activate the ABA signal transduction, and initiate the stress response, induce related functional genes to make various adaptive responses to alleviate the cellular damage.

## Conclusion

In conclusion, whether it is the phenotypic effect or the distribution and functional classification of differentially expressed proteins in the proteome, the physiological effect of ^12^C ion beam irradiation in the growth period of wheat seedlings is closer to that of ^60^Co-γ ray, but the effect is deeper at the same dose. However, the physiological effects of ^7^Li ion beam radiation treatment are quite different from the former, which are related to the different action principles of different types of heavy-ion beam radiation on organisms.

## Data Availability

The datasets presented in this study can be found in online repositories. The names of the repository/repositories and accession number(s) can be found in the article/Supplementary Material.
