# Supplementary material for: Physiological and Differential Proteomic Analysis at Seedling Stage by Induction of Heavy-Ion Beam Radiation in Wheat Seeds
Source: Front Genet. 2022 Jul 19;13:942806. doi: 10.3389/fgene.2022.942806 (PMC9343878; doi:10.3389/fgene.2022.942806)
Supplement: Supplementary file 1 [file DataSheet1.docx]

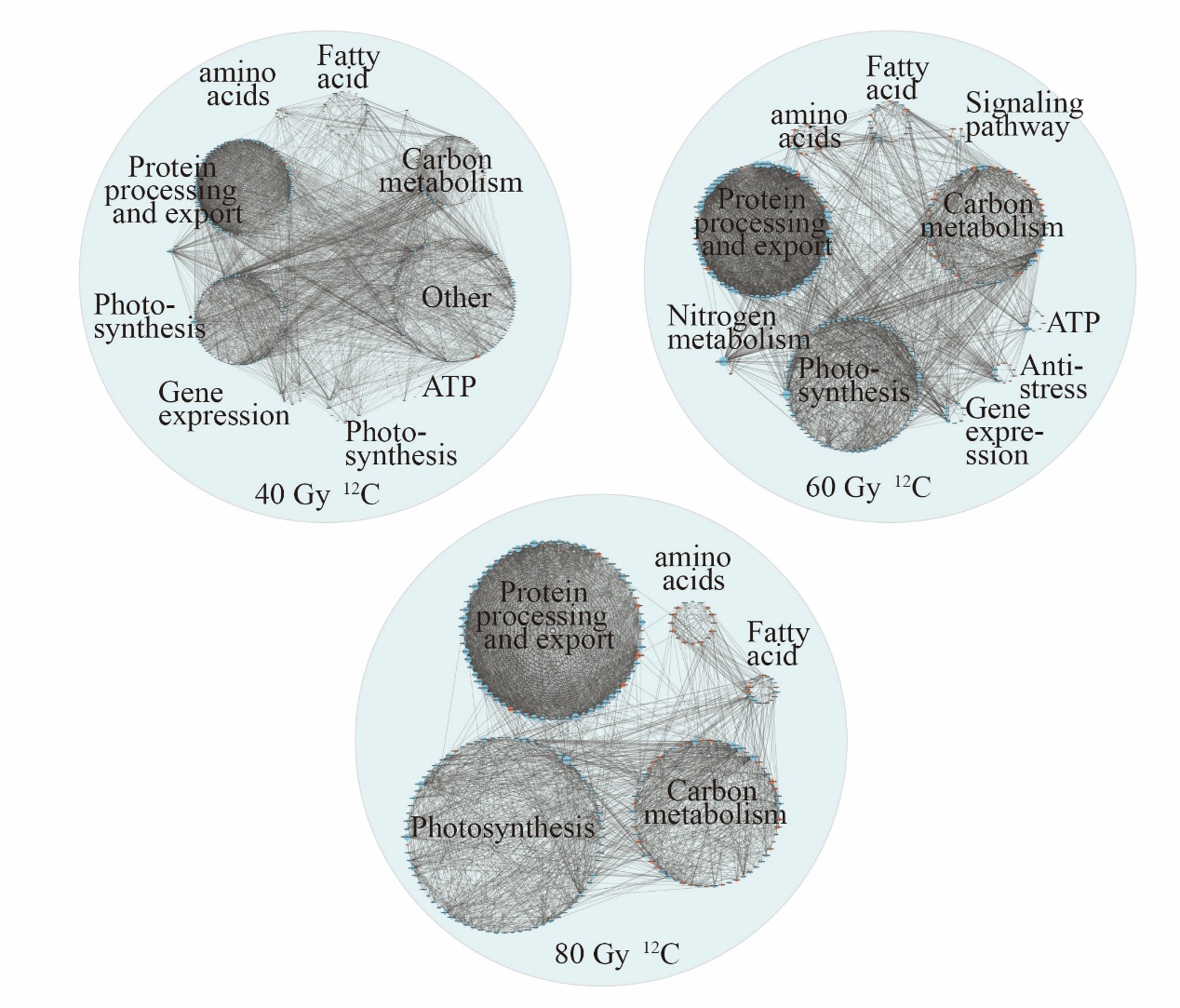


Supplementary Figure 1**|** Protein-protein interaction network for differentially expressed proteins


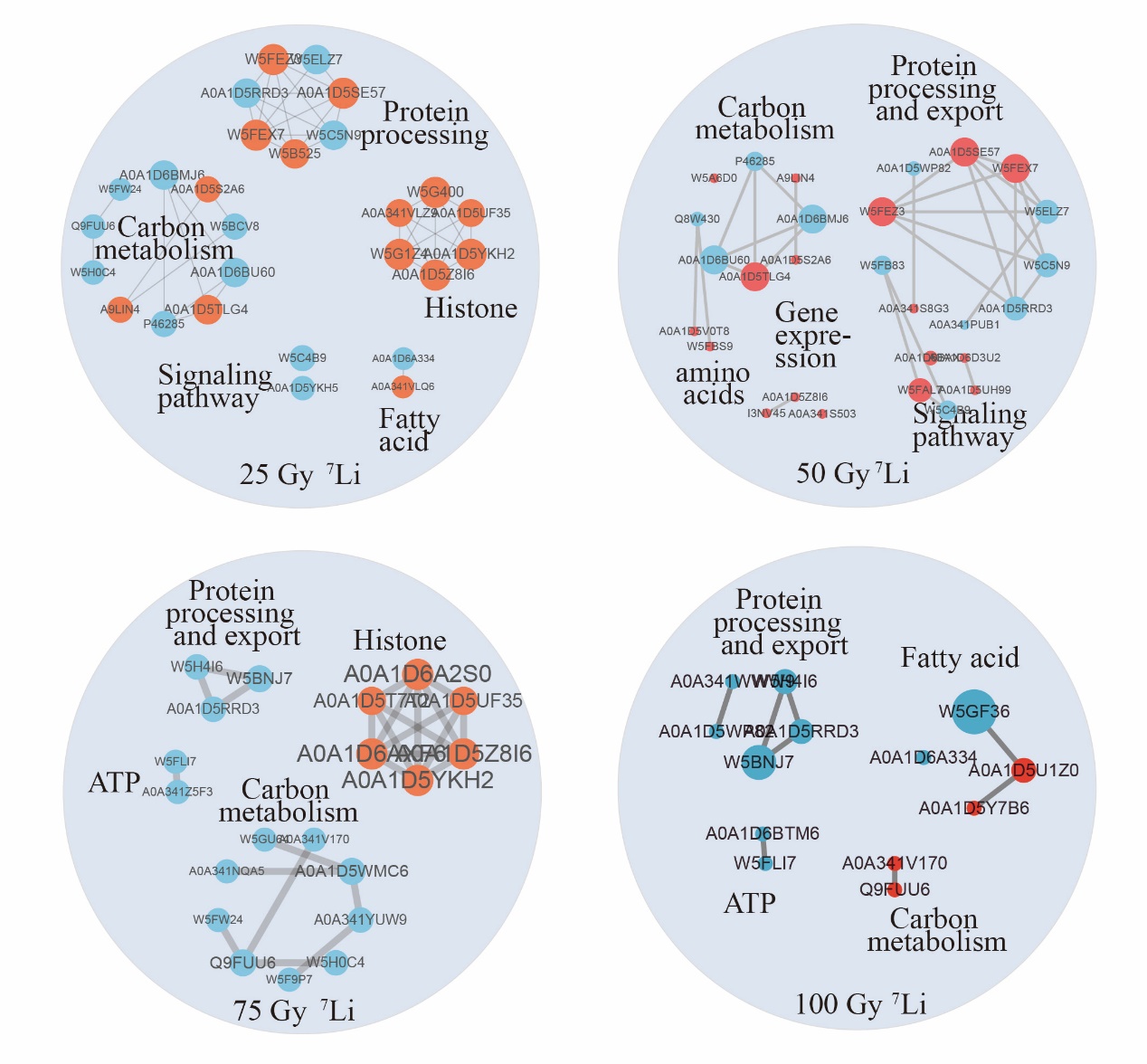


Supplementary Figure 2**|** Protein-protein interaction network for differentially expressed proteins


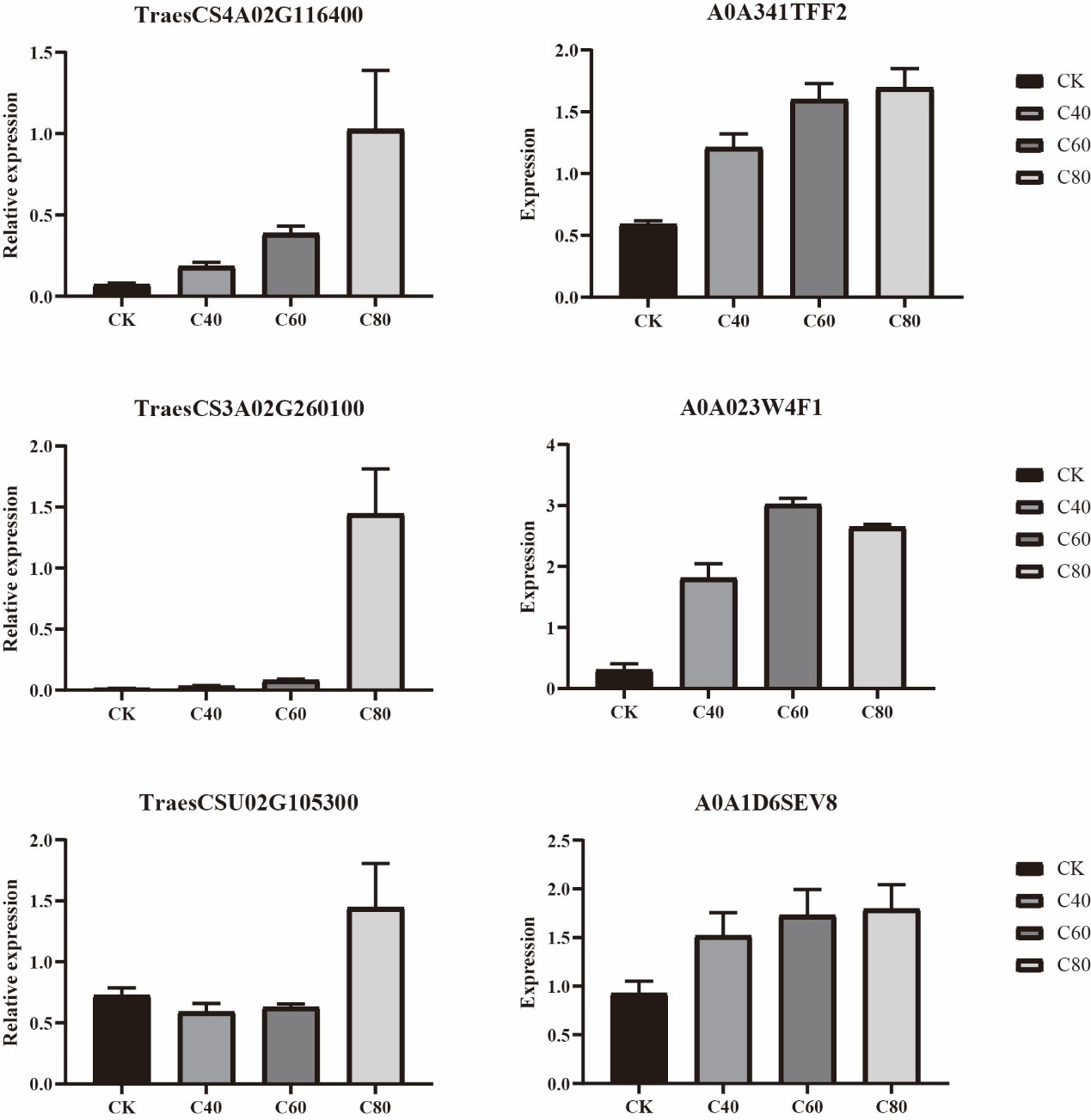


Supplementary Figure 3**|** qRT-PCR and protein quantification results
